# Religious Zeal, Affective Fragility, and the Tragedy of Human Existence

**DOI:** 10.1007/s10746-021-09575-6

**Published:** 2021-03-30

**Authors:** Ruth Rebecca Tietjen

**Affiliations:** Department of Communication, University of Copenhagen, Karen Blixens Plads 8, 2300 Copenhagen S, Denmark

**Keywords:** Religious zeal, Fanaticism, Affectivity, Passion, Human existence, Paul Ricœur

## Abstract

Today, in a Western secular context, the affective phenomenon of religious zeal is often associated, or even identified, with religious intolerance, violence, and fanaticism. Even if the zealots’ devotion remains restricted to their private lives, “we” as Western secularists still suspect them of a lack of reason, rationality, and autonomy. However, closer consideration reveals that religious zeal is an ethically and politically ambiguous phenomenon. In this article, I explore the question of how this ambiguity can be explained. I do so by drawing on Paul Ricœur’s theory of affective fragility and tracing back the ambiguity of religious zeal to a dialectic inherent to human affectivity and existence itself. According to Ricœur, human affectivity is constituted by the two poles of vital and spiritual desires which are mediated by the *thymos*. As I show, this theory helps us to understand that religious zeal as a spiritual desire is neither plainly good nor plainly bad, but ambiguous. Moreover, it enables us to acknowledge the entanglement of abstraction and concretion that is inherent to the phenomenon of religious zeal. Finally, this theory helps us to understand why religious zeal, as one possible expression of the human quest for the infinite, is both a promise and a threat. In conclusion, human existence is tragic not in that we necessarily fail, but in that no matter which path we take with regard to our spiritual desires—that of affirmation, rejection, or moderation—we are and remain fallible.

## Introduction

Today, in a Western secular context, the concept of religious zeal is often used synonymously with the concept of fanaticism (see critically Cavanaugh 2012; Olson 2007; Toscano 2017). Religious zeal is associated with religious intolerance, violence, and terrorism. In highlighting its allegedly inherent violent nature, public Western secular discourse tends to politicize and denigrate the phenomenon of religious zeal. Even if purely “private” forms of religious zeal are recognized—that is, forms of religious enthusiasm that remain restricted to one’s own life—they are nevertheless associated with a lack of rationality and autonomy and, therefore, viewed with suspicion (on fanaticism as a failure of rationality and autonomy, see Katsafanas 2019). Accordingly, the pejorative connotations surrounding the concept of religious zeal are based on both political and ethical considerations, with the latter concerning ideas of morality as well as of the good life.

The aim of this article is to put this denigrative view of religious zeal into question. To be sure, my description is guilty of making some unjustified generalizations. Religious zeal is not unequivocally identified with religious violence, nor is the pejorative view of religious zeal restricted to a Western secular context. However, such views are influential in this context—and they can, and indeed do, have devastating socio-political consequences. Thus, my analysis can more precisely be understood as an intervention into the public discourse that seeks to challenge the unambiguous identification of religious passion, intolerance, violence, and fanaticism. Although rhetorically hypostatizing a ‘we’ in talking about us as Western secularists, I am well aware that such an unambiguous ‘we’ does not exist.

The history of ideas points to a remarkable difference between the concept of fanaticism and that of religious zeal. The concept of fanaticism was introduced primarily to denote non-orthodox (orgiastic) religions. From the very outset, fanaticism had mainly negative connotations (Spaemann 1971). These negative connotations became intensified when, during the Reformation, the concept of fanaticism alongside those of enthusiasm and “Schwärmerei” became terms of political conflict, engaging combatants such as Luther, Locke, Hume, Shaftesbury, Voltaire, and Kant (Haas 2015; Heyd 2000; Klein and La Vopa 1998; Knox 1994; Spaemann 1971; Toscano 2017). By contrast, the concept of religious zeal traditionally denotes an ambiguous dimension of one’s own religion. For example, in Christianity, wrong, false, misguided, and blind forms of zeal are distinguished from true, godly, righteous, or holy ones (Edwards 2009: 352f.). Although this eclectic historical record does not provide a conclusive evidential basis on which to revise our conception of religious zeal, it still invites us to question our collective practice of denigrating religious zeal and to develop a more neutral understanding of what religious zeal is. This understanding will depend significantly on what we take religion to be and which religions and denominations we consider. Here, I will exclusively consider religious zeal in the context of Abrahamic monotheism and Christianity specifically. However, in doing so, I do not wish to deny either the existence of religious zeal outside of this context or differences within it.

The history of ideas does not call on us to simply neutralize our conception of religious zeal, but rather to be sensitive to the ethical and political ambiguity that is inherent to the phenomenon of religious zeal itself. Indeed, historical attempts to distinguish “problematic” forms of fanaticism from “innocent” forms of enthusiasm can be interpreted as an acknowledgment of the ambiguous role of religious (and non-religious) passions in politics and life in general. Religious zeal not only “fuel[s] militant religious and political conflicts with devastating social consequences, such as the Crusades, genocides, ethnic cleansings, terrorist attacks, and callous foreign policies,” it also animates “dedication to truth, love, and beauty” (McGregor 2006: 343). What is more, it also motivates political engagement for reconciliation, peace, liberty, equality, and justice (Appleby 2000).

To take an example, a widely admired pacifistic form of religiously inspired political engagement is embodied by Mahatma Gandhi and Martin Luther King Jr. More generally, it has been claimed that the realization of great ideas requires great passions, especially when these ideas challenge the existing social and political order (Walzer 2002). As the Protestant theologist Jonathan Edwards observes, “[t]wo things exceeding needful in ministers, as they would do any great matters, to advance the kingdom of Christ, are *zeal* and *resolution*. […] Most of the great things that have been done in the world of mankind, the great revolutions that have been accomplished in the kingdoms and empires of the heart, have been chiefly owing to these things” (Edwards 1970: 508f.). To be sure, religious zeal is not the only form of political enthusiasm that can bring about major political changes for the better (and the worse), and in concrete cases it may be contestable whether political actions are indeed religiously motivated. However, it is certainly one such form, and, at least in some cases, political enthusiasm is grounded in religious commitments. A historical example of political enthusiasm that was at least in part religiously motivated, and which from today’s perspective clearly enhanced democracy, is that of US-American Abolitionism (Olson 2007). This example illustrates the historical nature of our assessment of specific political manifestations of religious zeal; there is no “view from nowhere” when it comes to political judgments. But it also draws our attention to the ambiguous role of violence as a political means to bring about political change on the one hand and as inherent to the socio-political order on the other.

These observations invite us to reconsider our ethical and political evaluation of religious zeal. While religious zeal can give rise to political violence, it can also motivate nonviolent peacebuilding processes. It can be accompanied by morally blameworthy intolerance, a lack of rationality and autonomy, but it can also be an admirable form of wholehearted commitment to a righteous cause. Most notably, my examples illustrate that the ambiguity of religious zeal can be observed from both an internal and external perspective. It is explicitly acknowledged by religious traditions such as Protestant Christianity, and implicitly admitted by its sceptics who concede the value of political enthusiasm—even if religious in nature.

This gives rise to the question of how the ethical and political ambiguity inherent to religious zeal can be explained. Taking for granted that religious zeal is an affective phenomenon, we can approach this question in different ways. We can explore the ambiguous role of affects in the domains of ethics or politics (Nussbaum 2013; Walzer 2002), and we can reflect on the ambiguity of the sacred itself (Appleby 2000). In this article, I bracket these two questions and take a different approach, namely that of tracing back the ethical and political ambiguity of religious zeal to the dialectical character of human affectivity and existence itself. Doing so promises to deepen our understanding of the ambiguity of religious zeal itself, as well as that of the ambiguous role of affects in the domains of politics and ethics in general and the ambiguity of the sacred in particular. A more comprehensive approach would need to complement this analysis with a study of the other ambiguities mentioned above.

In tracing the ethical and political ambiguity of religious zeal to the dialectical character of human affectivity and existence itself, I draw on Paul Ricœur’s (1986) theory of affective fragility. Ricœur conceives of human affectivity as originally dual, consisting of vital and spiritual feelings. In doing so, his theory offers a counterpoint to many contemporary (analytic) theories of emotion that lack a comparable distinction and fail to acknowledge the ambiguity of human affectivity. This is the reason why I draw on Ricœur’s theory. Rather than taking it to be *true*—and making ontological commitments—I rely on it for *practical* reasons. It is a helpful tool because it offers us an understanding of human existence that allows us to account for the ethical and political ambiguity of religious zeal.

I argue, first, that by acknowledging spiritual feelings as one of two dichotomic poles of human affectivity, Ricœur’s theory of affective fragility helps us to account for the genuinely human nature of religious zeal and to conceive of it not as a pathological aberration but as an expression of the genuinely human quest for the unconditional, the total, the infinite. By uncovering the anthropological foundation of spiritual feelings, his theory neutralizes them. Second, I argue that Ricœur’s claim that within the sphere of spiritual feelings, the devotion to an idea and the loyalty to a ‘we’ are inextricably intertwined helps us to understand how within the phenomenon of religious zeal, abstraction and concretion—enthusiasm and love—are bound up with each other. Third and finally, I point out that Ricœur’s theory of affective fragility opens the possibility of a critique of human affectivity. With regard to spiritual feelings, three different failures can occur: we can suffer from an excess of spiritual desires; a lack, denial, or suppression thereof; or an appropriation of spiritual desires by excessive thymic desires for possession, power, or recognition (*Habsucht*, *Herrschsucht*, *Ehrsucht*). Yet, if it is true that not only the excess and lack of spiritual desires are potentially problematic but also their mediation through the *thymos*, then, for reasons I will explain later, human existence is tragic.

The structure of the paper is as follows. In the first section, I introduce the phenomenon of religious zeal. Drawing on my earlier research on religious zeal and contemporary (analytic) philosophy of emotions, I argue that religious zeal is an affective phenomenon that consists of wholehearted devotion to a transcendent religious object or idea of ultimate significance. In the second section, I introduce Ricœur’s theory of affective fragility. I embed this theory within the wider context of his theory of evil and interpret it against the backdrop of contemporary philosophy of emotions. In the third section, I situate the phenomenon of religious zeal within Ricœur’s theory of human affectivity and spell out how his theory can help us to gain a deeper understanding of religious zeal in general and the ethical and political ambiguity of religious zeal in particular.

## Religious Zeal

Religious zeal is an affective phenomenon. Affective phenomena are those that have a specific affective quality; it feels a specific way to be in the state in question. There are different types of affective phenomena, including bodily feelings, emotions, passions, and moods. As I have argued elsewhere, religious zeal can best be conceptualized as a passion (Tietjen 2021; see also Breyer 2020). Passions—or, as other people have called them, sentiments—are affective phenomena that consist of affective attachments to objects that are constitutive of the person’s practical identity and develop over time (on passions and sentiments, see Roberts 2007: 17–22; Ben-Ze'ev 2000: 82–86; Frijda et al. 1991; on practical identity, see Korsgaard 1996: 100–113; Frankfurt 1988c; Ricœur 1992: 113–139). They make us the person we are and endow our lives with continuity, coherence, and/or meaning. For example, my passion for poetry, my love for my family, my enthusiasm for philosophy, and my commitment to the idea of emancipatory liberation make me the person I am. If I lose or abandon them, I and others will have difficulties understanding myself (although, metaphysically speaking, I might still be the same person). Furthermore, my feeling of self-worth will be diminished, given that the attachments in question are descriptions under which I value myself. In being a passion—that is, an identity-defining affective attachment to an object that endows our life with continuity, coherence, or meaning—religious zeal differs from other affective phenomena such as the purely contingent, momentary desire for a lollipop.

As a passion, religious zeal is characterized by distinctive cognitive, affective, and motivational features. On the cognitive level, it involves the ascription of a final value. As in love, the object for which one is zealous is treated as valuable for its own sake rather than as only instrumentally valuable. Moreover, the evaluation is absolute. It cannot be relativized with reference to any prior or superior value. On the affective level, religious zeal is an attitude of wholehearted and unconditional commitment. On the motivational level, it is characterized by a threefold “uncompromisingness”. It necessarily expresses itself in behavior and actions. The actions in question are realized with all one’s power of will and energy. And zealots are willing to sacrifice in order to ensure the well-being of that to which they are affectionately attached, to realize what they are enthusiastic about, and to live up to the principles to which they are committed. As in all affective phenomena, the cognitive, affective, and motivational dimensions are inextricably interconnected (Goldie 2009: 50–83). The absoluteness of the value ascription is mirrored by the wholeheartedness of the zealot’s commitment. The wholeheartedness of the zealot’s commitment translates itself into the “uncompromisingness” of their actions. And the “uncompromisingness” of their action, in turn, points back to the absoluteness of the evaluation.

In being an identity-defining, wholehearted affective attachment to an object of ultimate significance that motivates uncompromising action, religious zeal resembles other passions such as moral or political enthusiasm. However, it differs from other passions insofar as the object of religious zeal is transcendent. In the case of Abrahamic monotheism, the object of religious zeal paradigmatically is God, but it may also be something like epiphany, eternal life, or the Kingdom of God (Breyer, 2020). Moreover, one might argue that religious objects are particularly well-suited as objects of zealous devotion because religion, by definition, involves a reference to the “holy” or “sacred” and, therefore, to the highest form of absolute value (see, for example, Otto 2010; Durkheim 2008). Likewise, one might point out that religious faith necessarily involves a discrepancy between epistemic evidence and degree of conviction. This not only makes religious objects particularly suitable as objects of passionate devotion, or so one might argue, but also gives religious zeal a specific flavor. This would imply that religious zeal differs from moral and political enthusiasm not just in being directed at a specific kind of object (paradigmatically God), but also in ascribing a specific kind of absolute value to its object (being holy or sacred), demanding a specific kind of wholehearted commitment (faith), and asking for a specific kind of uncompromising action (e.g., following divine commandments).

As a passion, religious zeal expresses itself in different emotions (on the co-constitution of passions and emotions, see Helm, 2001). For example, the zealot may feel joy or peace of mind when feeling close to God, or guilty when having transgressed divine commandments. Like passions, emotions are affective states of mind. However, emotions are not identity-defining affective attachments to objects that determine what we care about, what we attribute worth and value to. Rather, they are evaluative-representational states of mind that reflect *how it is going with* what we care about (Roberts 2003). As such, they differ from passions in that: first, they are bound to the specific situation we are in; second, they are usually less enduring than passions; and third, they are not necessarily identity-defining. Religious zeal not only *expresses* itself in a distinctive set of emotions, but is also partly *constituted* by them. Someone who claims to be zealous for God but does not show any affective reactions when what they are passionate about is doing well or badly, respectively, is not a religious zealot.

The emotions in which religious zeal characteristically expresses itself are “deep” (Vendrell Ferran 2019). Both the “uncompromisingness” of action and the depth of emotion that are characteristic of religious zeal mirror the absoluteness of the zealot’s evaluation and the identity-defining character of their affective attachment to a religious object of ultimate significance. The “depth” of an emotion is a measure of how integrated it is in the person’s web of mental states (Pugmire 2007: 30–77). As such, emotions that are based on identity-defining concerns are, by definition, “deep”. Moreover, it is not just the ultimacy and absoluteness of the zealot’s evaluation that demands uncompromising action. This “uncompromisingness” is also motivated by their desire to uphold their descriptive and evaluative (individual and collective) self-conception.

## Ricœur’s Theory of Affective Fragility

In the previous section, I introduced the phenomenon of religious zeal by conceptualizing it as an identity-defining, wholehearted affective commitment to a religious object of ultimate significance that motivates uncompromising action. In this section, I sketch Ricœur’s theory of affective fragility. As I explain in more detail below, Ricœur’s theory of human affectivity differs from other theories in that it conceives of human affectivity as something originally dual. This is why it is particularly suitable for understanding the ethical and political ambiguity of religious zeal. Thus, my use of Ricœur’s theory is pragmatically motivated. His theory helps us to understand an aspect of religious zeal that would otherwise remain unnoticed or harder to account for. Before outlining his theory, I situate it within his *Philosophy of Will*. This is a crucial step for understanding why and how a reflection on the affective fragility of man is relevant for understanding evil and, later on and more specifically, for understanding the ethical and political ambiguity of religious zeal.

Ricœur develops his theory of affective fragility in *Fallible Man* (1960). Together with *The Symbolism of Evil* (1960), it comprises the second volume of his *Philosophy of Will*, which deals with the problem of evil. The first part, *Fallible Man*, addresses the question of how evil is possible. It argues that the possibility of evil is grounded in the fallibility of man and that the fallibility of man can be traced back to the disproportion of the finite and the infinite that is characteristic of human existence (Ricœur 1986: 1–3; see also Kierkegaard 1983). More precisely, this disproportion is not a static feature of human existence, but rather speaks to the fact that we always already relate to these two poles.

According to Ricœur, a pre-philosophical understanding of the disproportion of human existence is given to us in the symbolic language of myths and the rhetoric of misery. These myths, in turn, are based on our “lived experience of ‘misery’” (Ricœur 1986: 83). The lived experience of misery, finally, essentially is an “immense and confused emotion”—that is, an affective state of mind (Ricœur 1986: 81). The pre-philosophical, mythological understanding of human nature is the starting point of both *Fallible Man* and *The Symbolism of Evil*. While the former uses the method of transcendental reflection, the latter uses (and develops) that of hermeneutics. The first part explores the anthropological foundations of evil. It addresses the question of how evil is possible. The second part is an investigation of the reality of evil. It explores the passage from innocence to guilt.

The analysis of the affective fragility of man in the fourth chapter of *Fallible Man* fulfills a double function. First, as an analysis of the disproportion of feeling, it complements the analyses of the disproportions of thinking and acting in the previous two chapters of the work. In this regard, it is on a par with the other two disproportions. Second, in our affectivity, the disproportion of human existence is experientially given to us in the form of conflict (Ricœur 1986: 91, 106f.). In this regard, an exploration of human affectivity is particularly apt for gaining a philosophical understanding of human existence, the disproportion of the finite and the infinite, the fallibility of man, and the possibility of evil. In the second and third chapters of *Fallible Man*, the plenitude of the lived experience and myths of misery had been intentionally bracketed for the sake of philosophical rigor (for this and the following, see Ricœur 1986: 5f., 81). In contrast, the mythological understanding of human existence sketched in the first chapter had been deep and total but remained pre-philosophical. The analysis of affective fragility as a lived experience of human misery promises to partly reintegrate the experiential and existential depth into the philosophical analysis and, therefore, to overcome the shortcomings of both the mythological preunderstanding and the transcendental analysis. Yet, this reintegration necessarily remains partial. The full depth of the mythical description is only accessible via hermeneutical interpretation, which is accomplished in *The Symbolism of Evil*. Therefore, the analysis of the affective fragility of man builds a bridge to the second part of Ricœur’s phenomenology of evil.

The two poles that, according to Ricœur, constitute the sphere of human affectivity are “vital feelings” (ἐπιθυμία) and “spiritual feelings” (ἔρως). From the perspective of contemporary philosophy and psychology of emotions, this is remarkable because rather than starting with the “simple,” “elementary,” and “basic” and only then proceeding to the “complex” and “composite”—as is characteristic of some naturalistic theories of emotions and, more specifically, theories of so-called “basic emotions” (Ekman 1992)—Ricœur acknowledges that human affectivity originally is dual and that only against the backdrop of this original duality can we understand genuinely human affects (Ricœur 1986: 92). As such, both feeling poles are originally neither morally good nor bad but neutral.

Vital and spiritual feelings are distinct in virtue of their conditions of fulfillment (Ricœur 1986: 93). While the fulfillment of vital feelings is supposed to be indicated by pleasure, the fulfillment of spiritual feelings would be indicated by happiness. The striving for pleasure represents the finite pole of human existence, the striving for happiness its infinite pole. Pleasure is finite because as the completion and perfection of “isolated, partial, finite acts, or processes,” it is a “provisory repose” (Ricœur 1986: 126, 93). The satisfaction that pleasure offers us is transient: “It dwells only in the instant, precarious and perishable like the very goods whose possession pleasure manifests in enjoyment” (Ricœur 1986: 94). Happiness, on the contrary, promises us “lasting peace” (Ricœur 1986: 93). It indicates “the perfection of the total work of man …, the termination of a destiny, of a destination or an existential project” (Ricœur 1986: 126). It is infinite because as a regulative ideal, it can never be realized: “no organized, historical community, no economy, no politic, no human culture can exhaust” our demand for totalization (Ricœur 1986: 103).

Both vital and spiritual feelings and pleasure and happiness are affective states of mind. However, they belong to different classes of affective phenomena. In terms of contemporary philosophy of emotions, vital and spiritual feelings can be understood as “concerns,” whereas pleasure and happiness resemble emotions. Concerns are affective attachments to objects to which we attribute importance, worth, or value (Roberts 2003: 141–151; see also Helm 2001: 67–75, on what he calls “focus”). Cognitive and conative dimensions thereby may be intertwined. In Ricœur’s theory, this entanglement is reflected by the fact that he categorizes vital and spiritual feelings as desires, but also discusses the evaluative properties of being lovable or hateful, desirable or loathsome, etc. (Ricœur 1986: 89). Emotions are evaluative, concern-based affective states of mind (Deonna and Teroni 2012; Goldie 2009; Helm 2001; Nussbaum 2001; Roberts 2003). They mirror how it is going with what we care about. *In this regard*, pleasure and happiness, as the affective states that indicate the fulfillment of our vital and spiritual desires, respectively, resemble emotions. Although controversial in philosophy of emotions, one might even say that pleasure *is* an emotion (Helm 2002). In the case of happiness, however, things are less straightforward. One of the key features of happiness is that it transcends our momentary situation and fragmented concerns. Ricœur thus reminds us that there might be affective states that resemble emotions in being evaluative, and yet resemble moods in reflecting how it is going with our life—or life—*in general* rather than just with a particular concern (of a particular person) at a given moment of time (Rosfort and Stanghellini, 2009).

Spiritual feelings hence reflect the human demand for totalization. We are not just striving for pleasure, the fulfillment of isolated, partial, and finite desires, but are also concerned with the question of what it means to lead a good life (Ricœur 1986: 101–106). This in turn involves a reflection not only on our personal life but also on our society and the world at large. Happiness as the feeling that would indicate the fulfillment of our spiritual desires mirrors this demand in reflecting how it is going with (our) life in general, rather than reflecting how it is going with a particular concern of ours at a given moment of time. Drawing on ancient philosophy and the Kantian idea of happiness (in proportion to virtue) as the highest good (Kant 1908: 110f.), Ricœur points out that the striving for happiness mirrors, on the level of feeling, reason’s demand for totality—or the other way around: reason’s demand for totality renders more clearly our affective demand for happiness.

It is not only the (lack of) fulfillment of vital and spiritual desires that expresses itself in affective states of mind; the conflict between vital and spiritual desires does, too. As Ricœur points out, it is distinctive of the disproportion of feeling (as opposed to the disproportions of thinking and acting) that it is an *interiorized* disproportion: a disproportion that is felt as conflict (Ricœur 1986: 106f., 131f.). This potentially conflicting nature of our affects is what makes us fragile.

The conflicts between vital and spiritual desires point us to the mediating sphere of the *thymos* (ϑυμός). While vital feelings do *not yet* distinguish different people from each other, spiritual feelings point *beyond* our individuality by referring to the idea of being part of something greater, a community or an idea (Ricœur 1986: 107). Accordingly, it is the *thymos* in which the human self is constituted. Ricœur distinguishes three kinds of thymic desires: the desire for possession, power, and recognition. They are located in three different spheres that (like the vital and the spiritual) are constitutive of human existence: the economic, political, and cultural sphere. Even if, empirically speaking, they are primarily given to us as aberrations, thymic desires are originally neither morally good nor bad (Ricœur 1986: 111f.). While the termination of vital feelings expresses itself in pleasure and the termination of spiritual feelings would express itself in happiness, the termination of the thymic desires is undetermined (Ricœur 1986: 127). It is unclear what amount of possessions, power, and recognition are *enough*, which is why they do not give rise to a provisory repose or lasting peace, but by their very nature are “restless” (Ricœur 1986: 126). This indefiniteness is characteristic of all human actions and human existence as such. The desires for possession, power, and recognition reflect our intersubjective nature; they reflect the fact that whatever we encounter in the world is never simply “natural” or “spiritual” but always infused with intersubjective meaning (Ricœur 1986: 112). Accordingly, in humans, vital and spiritual desires never occur in a pure state. They are always commingled and infused with thymic desires.

As emphasized at the outset, what is remarkable about Ricœur’s theory of human affectivity is that rather than starting from something simple and then proceeding to the complex, Ricœur starts from an original polarity. This invites a reconsideration of the widespread assumption that the primary distinction to be drawn in the field of emotions is that based on the latter’s “formal objects”. According to contemporary philosophy of emotions, formal objects are evaluative properties that individuate emotion types (Deonna and Teroni 2012: 40–42; Teroni 2007). For example, “hope … holds the obstacle as proportioned to my forces and the good as accessible; despair … makes the good seem out of reach; fear … feels evil to be superior to my forces and invincible” (Ricœur 1986: 108). Although Ricœur acknowledges the importance of such distinctions, he points us to another, equally fundamental typology: a typology based on the question of which ontological sphere the objects of our desires, emotions, and moods belong to. Accordingly, conflicts arise not only because, for contingent reasons, our desires are not compatible with each other; they also reflect a more fundamental tension grounded in human existence itself. As I show in the next section, it is the acknowledgment of this tension that makes Ricœur’s theory particularly suitable for accounting for the ambiguity of religious zeal.

## The Tragedy of Human Existence

In the previous two sections, I introduced the phenomenon of religious zeal and Ricœur’s theory of affective fragility. In this section, I turn to the question of how Ricœur’s theory helps us to understand the phenomenon of religious zeal and make sense of its ethical and political ambiguity.

### Totalization and the Promise of Happiness

To start with, Ricœur’s theory of human affectivity helps us to “neutralize” religious zeal or even to appreciate it as *one* expression of *one* pole—the infinite pole—of human existence itself. That is to say, if we bracket its concrete historical manifestations, religious zeal is ethically and politically neither good nor bad but reflects a specific human need, namely the need for the unconditional, infinite, and absolute. I introduced religious zeal as a passionate phenomenon. Passions are specific kinds of concerns, namely such that are constitutive of a person’s identity. In Ricœur’s terminology, religious zeal, then, must be classified as a desire; it is directed at transcendent religious ideas and is, therefore, a spiritual rather than a vital desire. As I have shown, in Ricœur’s framework, the classification of desires as spiritual is based on their fulfillment conditions. Spiritual desires are those whose fulfillment would be indicated by happiness. However, as the regulative ideal of practical reason, happiness can never be fully achieved. Whereas bracketing the concrete forms in which religious zeal historically manifests itself allows us to appreciate religious zeal as one possible expression of the human quest for the infinite, in reality, religious zeal always already takes a specific form and realizes its destructive and/or constructive potential. In this sense, it is more appropriately described as ambiguous rather than neutral.

Ricœur’s theory also helps us to understand the totalizing tendency of religious zeal as inherent to human nature as such. As I have shown above, following Ricœur, vital feelings are isolated, partial, and finite needs whose fulfillment promises us a provisory repose. Spiritual feelings, by contrast, reflect the human demand for totalization whose fulfillment promises us lasting peace. Although all forms of religious zeal involve a reference to something absolute, they still occur in different degrees of totalization (Tietjen 2021; for a similar yet conceptually different differentiation, see Katsafanas 2019; Passmore 2003). Totalization occurs on two axes. The first is that of domains of life. Some zealots conceive of religion as one domain of their lives among others. The “totalization” in this case is restricted to these religious domains of life in which they worship the holy. Other zealots conceive of religion as the form of their life—that is, as an overarching principle that determines the meaning and goal of their life as a whole. The second axis concerns the question of to whom the zealots apply their principles. Some people zealously devote their own lives to a religious idea without showing the same form of rigidity with regard to the lives of others, be it because they take the meaning and goal of human life to be something individual—a matter of personal choice or destiny, for example—or because they do not take themselves to be in the position to judge the lives of others. Others take their religious goals to determine the meaning and goals of mankind as such. This second axis of totalization introduces a distinction between individual and collective conceptions of happiness—collective conceptions of happiness that, as such, necessarily involve conceptions of morality and politics or the political.

Cultural anthropologists, such as Jan Assmann, have pointed out that historically the totalizing tendency of religion is closely related to the emergence of Abrahamic monotheism (Assmann 2018). Drawing on the Kantian idea of happiness in proportion to virtue as the highest good, Ricœur, however, notes that the totalizing tendency of religious zeal can also be understood as inherent to human reason, action, and affectivity itself (Ricœur 1986: 101–106). In doing so, Ricœur neutralizes the idea of totalization along both axes and helps us to appreciate them as an expression of human nature. Developing a sense of what is good not only for us but for the world at large is a demand characteristic of human existence itself. Importantly, this demand for totalization is just as fundamental as the opposite demand for particularization that is represented by vital feelings.

On the personal level, *the very fact of having* objects in our lives to which we are wholeheartedly devoted—people we love, ideas we are enthusiastic about, values we are committed to—is something we prima facie appreciate (Frankfurt 1988b, 1988c, 2004). Moreover, not only *having objects in our lives* we are wholeheartedly devoted to, but also wholeheartedly devoting *our lives* to a (group of) person(s) or idea(s) is an object of social admiration or at least fascination. This is particularly evident if we consider non-religious forms of devotion such as the vocation of an artist, scientist, philosopher, caregiver, or medical professional to their respective profession. Of course, for our overall judgment, it matters whether the objects in question are indeed *worthy* of wholehearted devotion and, especially, wholehearted devotion *of one’s whole life*. We may admire romantic lovers for their attempt to become one with their beloved but still be worried about their depoliticized form of life. We may admire scrupulous investment bankers for their passionate devotion but still criticize them for mistakenly treating money as the only value there is. Especially, moral questions matter. Our sympathy for the football supporter’s fandom is bound to the condition that they do not become a violent hooligan. We may wonder whether it is indeed appropriate to admire artists or philosophers for their devotion to their profession although they misuse their positions of power and neglect their social responsibilities to their students. Finally, we may blame pick-up artists who zealously devote their lives to becoming the most efficient seducers for their sexism. But still, *prima facie* there is something appealing about wholehearted devotion in general and wholehearted devotion to *one* object (person or idea) in particular. The risk—and the promise—thereby increases with the degree of totalization and personal investment.

Given these considerations, as Western secularists, we may be inclined to concede that religious zeal, under certain conditions, indeed can be valuable so long as it remains clearly restricted to one’s own life—to admire or at least tolerate, in other words, the monk in the desert but not the religiously motivated political activist. Indeed, we may say that on the second axis of totalization, mystics tend to fall into the first category, while fanatics tend to fall into the second one. Mystics focus on their own conduct, while—according to a common prejudice—they are not necessarily interested in the lives of others or the structures of our society as a whole. Fanatics, by contrast, tend to focus on the conduct or, more precisely, *failures* of others, while losing sight of their own failures and fallibility, the atrocity of their actions, and even worse: conceiving of their atrocities as a condition of both their own salvation and that of mankind.

However, the juxtaposition of unpolitical mystics and intolerant violent fanatics is sorely incomplete. It ignores political mysticism and nonviolent, religiously motivated political engagement as symbolized by the figures of the mystic or the saint who, through their own exemplification of virtue, become sources of inspiration for others and society as a whole (see, for example, Bergson 2002: 209–265; Underhill 1990: 413–443). Ricœur’s theory of human affectivity reminds us that an understanding of the highest good is only complete if it involves both an individual conception of happiness and a collective conception of morality, virtue, or justice—an understanding not only of how we as individuals are supposed to live our lives, but also of how we as social beings are supposed to live together and, even more generally, what the world we inhabit together is supposed to look like. Not every totalization is totalitarian—although in being total, it bears the danger of becoming so.

Moreover, obviously, not every totalitarianism is religious—although in involving totalization, it resembles religion. Indeed, the concept of “political religion” was invented as an analytic tool to help us understand the nature and success of totalitarianism (Gentile, 2006). It captures the idea that political phenomena such as Russian Communism, Italian Fascism, and German National Socialism resemble religion in their display of typical elements of traditional religions. Since religion is a multidimensional phenomenon involving experiences and affects, doctrines and beliefs, ritualized practices and actions, communities and institutions, the quasi-religious character of totalitarian movements can be sought in different spheres. For present purposes, one key feature of political religions is the fact that they offer us a definition of “the meaning and finality of human existence” (Gentile 2005: 29). More precisely, they assert “in an *exclusive* and *complete* way … the prerogative to define the *ultimate* meaning and the *fundamental* goal of human existence on earth” (Gentile 2005: 29, my emphasis). Even if these goals are qualified as “goals of human existence *on earth*,” their claim to totality, ultimacy, and fundamentality still point to the fact that they function as regulative ideals and the fact that they do so is part of what lends the political movement an “aura of sacredness” (Gentile 2005: 29, my emphasis). Both “political religions” and religious zeal thus essentially involve (or are) spiritual desires. However, it is important to note that the concept of political religion is not just an analytic tool. It was invented for *critical* purposes. According to its inventors, political religions are *perverted* forms of religion whose success is directly linked to modernity and secularization (Gentile 2005: 26). So, according to this picture, totalitarianism does not prove the violent nature of religion (although it still might be interpreted as evidence for its violent potential).

### Concretization and the Willingness to Sacrifice

Ricœur’s theory of human affectivity draws our attention not only to the fact that religious zeal resembles and can take the form of *political* devotion, but also to the fact that religious zeal resembles and indeed involves forms of *personal* devotion—that is, forms of devotion to a person or group of persons, such as friendship, love, or comradeship.

Within spiritual feelings, Ricœur distinguishes two kinds of feelings: feelings relating to “interhuman participation in various forms of ‘We’” and feelings relating to the “participation in tasks of supra-personal works that are ‘Ideas’” (Ricœur 1986: 103–105). As spiritual desires, both kinds of desires aim at happiness and in aiming at happiness they are infinite. Personal forms of devotion range from highly personalized ones like romantic love to highly collective ones like the populist’s commitment to “the people”. Moreover, as spiritual desires, personal forms of devotion involve a totalizing tendency. Exemplarily, this totalizing tendency can be observed in the romantic lover’s attempt to become one with their beloved and the populist’s mystification of “the people” as a promise of immanent salvation (Laclau 2007).

Religious zeal resembles person-directed forms of devotion in that both are spiritual desires that aim at happiness and involve a totalizing tendency. While the religious zealot aims at happiness through the realization of a specific idea, the romantic lover aims at happiness through becoming one with a beloved person. At first sight, the objects of the two classes of spiritual desires distinguished by Ricœur seem to belong to two different ontological categories: the abstract and the concrete. In terms of affects, we can distinguish two idealized types of affects corresponding to these spheres: enthusiasm and love. While enthusiasm is directed at something abstract—an idea, a value, a principle—love is directed at something concrete—a person, a people, a place. While love aims at the flourishing, well-being of, and union with what one loves, enthusiasm aims at realizing the idea one is enthusiastic about.

However, as we learn from Ricœur, both kinds of feelings—enthusiasm and love—are always already reciprocally entangled. “It is always an Idea that gives a horizon of meanings to the growth of a ‘We’ and a ‘friendship’” (Ricœur 1986: 103). What Ricœur suggests here is that groups (of two or more people) are partly constituted by shared commitments to values and ideas and that, even more relevant for our present purposes, values and ideas always already are and need to be embodied by specific persons, places, objects, or symbols in order to become an object of passionate devotion (Laclau 2007).

Indeed, religious zeal not only resembles but essentially *involves* personal forms of devotion. For example, the idea of God or the Holy is not just an abstract idea; it is always specific in that it differs from ideas of God or the Holy that can be found in other denominations and religions, times and places. Even if the religious zealot is a lonely ascetic—or the religious fanatic a lone wolf—their identity as religious zealot or fanatic, respectively, is still in part socially constituted. It is determined by the (real or imaginary) religious community to which they take themselves to belong (as well as by “the others”—us?—who observe the spectacle). Even more obviously, other forms of religious zealotry and fanaticism involve references not only to mythical and imagined but also real communities of faith, fellow believers, and religious leaders.

Exemplarily, the dialectic of concretion and abstraction inherent to the phenomenon of religious zeal is expressed in the discussion in Christian theology over whether religious zeal is to be understood as a form of zealotry or jealousy—words that, not accidentally, have the same etymology. The Hebrew concept of zeal, קנִאְָה (qin’â), which is used in the Old Testament to describe both human zeal and God’s zeal, can mean both anger (or wrath) and jealousy. As such, the contested question of how the concept in specific passages is to be translated is of both linguistic and systematic interest. It points us to two different ways in which the relationship between God and the people of Israel can be construed (Assmann 2018). Taking for granted that anger is a reaction to the violation of a moral norm (Pettigrove 2012), the conceptualization of religious zeal as an anger-like emotion represents the underlying relationship as passionate commitment of the people of Israel to the divine commandments and, therefore, as what I have called enthusiasm. By contrast, the conceptualization of religious zeal as a form of jealousy represents the relationship as a love-like (reciprocal) relationship between the people of Israel and God that (like traditional romantic love) demands exclusivity (on jealousy, see Farrell 1980; Kristjánsson 2016; see also Ricœur 2010: 35).

The shared nature of abstract and concrete forms of devotion, according to Ricœur, is most vividly visible in the phenomenon of sacrifice:

[S]acrifice attests that, at the limits of life, to give one’s life for a friend and to die for an idea is the same thing. Sacrifice shows the fundamental unity of two schemata of belonging, the schema of friendship and the schema of devotion (or loyalty). Friendship is to another what devotion is to an idea, and the two together make up the view – the *Aussicht *– ‘into an order in which, alone, we can continue to exist’. (Ricœur, 1986: 104)

This insight is remarkable because it attests that sacrifice—and, with it, violence—is not (only) an aberration but (also), as an expression of human transcendence, marks the border of spiritual desires themselves. Only if we take this seriously can we understand the tragedy of human existence that results from the fact that we cannot know which exceptional situations allow or demand sacrifice.

### Toward an Ethics of Affects

As outlined above, in *Fallible Man*, Ricœur uses a transcendental method to develop a theory of human nature. In this regard, his work is primarily descriptive. However, his main concern is to understand human evil. He develops a theory of human nature in general and of human affectivity in particular in order to explore the question of how evil is possible—why we are fallible. Therefore, *Fallible Man* implicitly—and to some extent explicitly—involves reflections on the question of what this failure may look like from the perspective of affective psychology. This allows us to develop an ethics of affects. Rather than evading conflict by dissolving it in favor of one or the other side and providing a clear answer to the question of what is right and wrong, this ethics takes the potentially conflicting nature of our desires seriously and asks us to acknowledge our own fallibility.

First, failure may occur when one of the two poles of human affectivity becomes excessive. An excess of spiritual desires consists of *wrongly* sacrificing one’s life (or the lives of others), wealth, power, or recognition (or those of others) for a friend, a community, or an idea. An excess of vital desires, by contrast, consists of *wrongly* sacrificing one’s friends, comrades, or ideas for our life. Thymic desires can become excessive, too. We can *wrongly* sacrifice our life, our friends, comrades, or ideas for wealth, power, or recognition.

The willingness to sacrifice—to, in extreme cases, give one class of desires absolute precedence over the others—is inherent to the logic of each of the three classes of desires itself. It is grounded in, first, the kind of fulfillment they promise us and, second, the way in which they constitute conditions of our other desires. The willingness to sacrifice is inherent to the logic of vital desires because, first, in promising us pleasure, they promise us perfection. Even if perfection is a finite form of fulfillment, it can still be treated as absolute. Second, in promising us (finite) perfection, vital feelings remind us that “living is not one activity in the midst of others but the existential condition of all others” (Ricœur 1986: 94). Accordingly, even if our life is not a condition of the existence and realization of values, it is still a condition of *us* valuing and realizing values. As outlined in the previous section, the willingness to sacrifice is inherent to the logic of spiritual desires because they promise us infinite perfection. In doing so, they remind us of our transcendent nature and of the fact that our life only makes sense if we are part of something greater. Accordingly, even if friendship or being part of a group and being devoted to an idea are not conditions of life as such, they are still conditions of a meaningful life. The thymic desires, finally, involve a tendency to sacrifice because by their very nature they are restless and tend to ask for *more*: more money, more power, more recognition. Without a predefined limit, they harbor the possibility of demanding the subordination of all other desires to them.

To summarize, Ricœur’s theory helps us to understand that the willingness to sacrifice characteristic of religious zeal—for good and bad—reflects the nature of spiritual desires and human existence as such. Each of the three classes of desires promises us a different form of perfection that may be interpreted as, in extreme cases, overriding any other demand. We are fallible in that we cannot know which exceptional situations allow or demand sacrifice. There is no “neutral” point of view from which we can decide this question.

Second, both the excess and the lack or suppression of spiritual desires can give rise to failure. To deny spiritual desires is to deny an important aspect of human existence, namely its infinite or transcendent nature. Criticizing religious zealots for their zealotry based *solely* on the infinite nature of their desires (or the religious character of the answers they seek to their infinite desires) is, therefore, problematic and, more importantly, dangerous. It may make us blind to the dangers of searching for happiness in pleasure, money, power, or recognition alone and—even worse—to our own implicit (secular) claims to the unconditional with reference to which we justify our own violence in the guise of secular reason (Staudigl 2020).

If both the excess and the lack of spiritual desires are dangerous, we might be inclined to seek salvation in moderation. And indeed, the whole idea of the *thymos* is that of mediation between our vital and spiritual desires. However, mediation is not identical with moderation; it just means that it is not some desires that we simply find in ourselves that determine our actions, but *we ourselves* who have the capacity to relate to them—to embrace, deny, or moderate them (Frankfurt 1988a). And moderation itself can be problematic. Even if exceptional, spiritual desires in some cases may demand sacrifice (and, again, we cannot know for sure which cases these are): “one cannot compromise over the holy without compromising the holy” (Margalit 2009: 24). Therefore, third, in our moderation we are as fallible as in our enthusiasm and love.

Fourth and finally, failure may occur due to an inappropriate appropriation of our spiritual feelings through our thymic desires for power, possession, and recognition. As argued above, in human beings, spiritual desires never occur in a pure form; they are always already infused with vital and thymic desires. Again, it is thereby not the entanglement itself that is problematic, but the entanglement comes along with a specific kind of fallibility. Exemplarily, this kind of fallibility is expressed in the critical question of whether a specific (group of) fanatic(s) *really* is driven by religious concerns or whether their *real* motive is a much more mundane desire for possession, power, and recognition.

Ricœur’s theory of affective fragility thus helps us to understand both the promise and the threat of religious zeal as a passionate form of devotion to a religious object of ultimate significance that expresses itself in uncompromising actions. As a transcendental analysis, it provides no answer to the moral question of *when* a specific expression of religious zeal is morally or politically praise- or blameworthy, nor to the question of *why* some (religious) zealots—some of *us*—fail more often or more badly than others. All that his theory offers us is a deepened understanding of the fact of our fallibility. Human existence is tragic not in that we necessarily fail, but in that no matter which path we take with regard to our spiritual desires—that of affirmation, rejection, or moderation—we are and remain fallible.

Against the backdrop of this analysis, the unambiguous identification of religion, passion, and violence that is characteristic of public, Western, secular discourse can be interpreted as a denial of fallibility or, more precisely, of *our own* fallibility—the fallibility of the standpoint of finite secular reason. Although the refutation of the claim that religious zeal necessarily comes with a lack of reason, rationality, and autonomy demands further argumentation, my analysis demonstrates why the unambiguous identification of religion, passion, and violence is both wrong and dangerous. Rather than denying (or externalizing) fallibility, we should acknowledge it as a basic feature of human existence itself that reflects the potentially conflicting nature of our desires. Conflicts thereby arise not only because, for contingent reasons, our desires are not compatible with each other; they also reflect a more fundamental tension grounded in human existence itself. Recognizing this not only helps us to acknowledge the ethical and political ambiguity of religious zeal. It also has far-reaching consequences for our understanding of the complex relationship between religion, passion, violence, and politics in general. The exploration of these consequences, however, must be left open for future research. In his late article on religious belief, Ricœur (2010: 39) himself suggests that the acknowledgment of human fallibility in the domain of religion in the most advanced stage might result in a deep form of humility. We may wonder, however, whether this form of humility is still compatible with religious zeal.
